# Maternal Folate Status and the BHMT c.716G>A Polymorphism Affect the Betaine Dimethylglycine Pathway during Pregnancy

**DOI:** 10.3390/nu8100621

**Published:** 2016-10-09

**Authors:** Jose M. Colomina, Pere Cavallé-Busquets, Sílvia Fernàndez-Roig, Pol Solé-Navais, Joan D. Fernandez-Ballart, Mónica Ballesteros, Per M. Ueland, Klaus Meyer, Michelle M. Murphy

**Affiliations:** 1 Area of Preventive Medicine and Public Health, Faculty of Medicine and Health Sciences, Universitat Rovira i Virgili, IISPV, C/Sant Llorenç 21, Reus 43201, Spain; josemaria.colomina@urv.cat (J.M.C.); silviafroig@gmail.com (S.F.-R.); pol.sole@urv.cat (P.S.-N.); joan.fernandez-ballart@urv.cat (J.D.F.-B.); 2 Ciberobn Fisiopatología de la Obesidad y Nutrición (CB06/03), Instituto Carlos III, Madrid 28029, Spain; pecavalle@grupsagessa.com; 3 Area of Obstetrics and Gynaecology, Hospital Universitari Sant Joan, Reus and Universitat Rovira i Virgili, Reus 43204, Spain; 4 Area of Obstetrics and Gynaecology, Hospital Universitari Joan XXIII, Tarragona and Universitat Rovira i Virgili, Tarragona 43005, Spain; mobape71@yahoo.es; 5 Section for Pharmacology, Department of Internal Medicine, University of Bergen, Bergen N-5020, Norway; per.ueland@ikb.uib.no; 6 Bevital A/S, Laboratory building, 9th floor, Bergen N-5021, Norway; klaus.meyer@bevital.no

**Keywords:** pregnancy, folate, betaine: homocysteine methyltransferase, BHMT c.716G>A, betaine, dimethylglycine

## Abstract

The effect of the betaine: homocysteine methyltransferase BHMT c.716G>A (G: guanosine; A: adenosine) single nucleotide polymorphism (SNP) on the BHMT pathway is unknown during pregnancy. We hypothesised that it impairs betaine to dimethylglycine conversion and that folate status modifies its effect. We studied 612 women from the Reus Tarragona Birth Cohort from ≤12 gestational weeks (GW) throughout pregnancy. The frequency of the variant BHMT c.716A allele was 30.8% (95% confidence interval (CI): 28.3, 33.5). In participants with normal-high plasma folate status (>13.4 nmol/L), least square geometric mean [95% CI] plasma dimethylglycine (pDMG, µmol/L) was lower in the GA (2.35 [2.23, 2.47]) versus GG (2.58 [2.46, 2.70]) genotype at ≤12 GW (*p* < 0.05) and in the GA (2.08 [1.97, 2.19]) and AA (1.94 [1.75, 2.16]) versus GG (2.29 [2.18, 2.40]) genotypes at 15 GW (*p* < 0.05). No differences in pDMG between genotypes were observed in participants with possible folate deficiency (≤13.4 nmol/L) (*p* for interactions at ≤12 GW: 0.023 and 15 GW: 0.038). PDMG was lower in participants with the AA versus GG genotype at 34 GW (2.01 [1.79, 2.25] versus 2.44 [2.16, 2.76] and at labour, 2.51 [2.39, 2.64] versus 3.00 [2.84, 3.18], (*p* < 0.01)). Possible deficiency compared to normal-high folate status was associated with higher pDMG in multiple linear regression analysis (β coefficients [SEM] ranging from 0.07 [0.04], *p* < 0.05 to 0.20 [0.04], *p* < 0.001 in models from early and mid-late pregnancy) and the AA compared to GG genotype was associated with lower pDMG (β coefficients [SEM] ranging from −0.11 [0.06], *p* = 0.055 to −0.23 [0.06], *p* < 0.001). Conclusion: During pregnancy, the BHMT pathway is affected by folate status and by the variant BHMT c.716A allele.

## 1. Introduction

The importance of one carbon metabolism and homocysteine regulation in foetal development and optimal pregnancy outcome is well established. Homocysteine remethylation contributes not only to homocysteine homeostasis but also to the provision of methyl groups essential for foetal development. Homocysteine is remethylated to methionine by the ubiquitous folate and cobalamin-dependent methionine synthase (MTR; EC 2.1.1.13) and by betaine:homocysteine methyltransferase (BHMT; EC 2.1.1.5) that occurs mainly in the kidney and liver [1]. The estimated consumption of homocysteine by MTR and BHMT is similar in rat liver [2] but globally the MTR reaction is thought to prevail in mammals based on the fact that BHMT activity has only been detected in some organs such as the liver and kidney [3,4] with conflicting reports regarding its presence in the brain [1,5,6]. To the best of our knowledge this information is not available in human studies to date but a study of healthy Dutch adults reported that the inverse association between folate and fasting plasma total homocysteine (tHcy) was stronger than that between betaine and tHcy, suggesting that the MTR reaction prevails under normal circumstances [7]. Some studies also provide indirect evidence for interaction between both remethylation pathways and upregulation of the BHMT pathway when folate status is low [8,9,10,11,12]. Serum folate was positively associated with betaine and inversely associated with dimethylglycine in a study of healthy Dutch adults [8] and serum dimethylglycine was higher in folate-deficient compared to normal status adults in a USA study [9]. Stronger negative associations between betaine and post-methionine load tHcy were observed when serum folate status was low [10] or in the absence of B vitamin supplement use [11]. We reported an apparent shift in the roles of folate and betaine in homocysteine homeostasis as folate status declines with advancing pregnancy and that low plasma folate status was associated with low plasma betaine and high dimethylglycine and a high dimethylglycine/betaine ratio [12].

BHMT c.716G>A (also known as 742G>A, rs3733890) is a common single nucleotide polymorphism (SNP) in which arginine is substituted by glutamine at position 239 [13]. Numerous human studies reported no effect of the SNP on tHcy [14,15,16,17,18]. However, a Norwegian study did report that plasma dimethylglycine decreased with increasing number of A alleles [17].

The BHMT c.716G>A SNP in pregnant women has been associated with increased risk of placental abruption [16]. There are conflicting reports of the effect of the SNP in the foetus on foetal development outcomes. The variant allele was associated with increased risk of grave neural tube defects (NTDs) [19,20,21] but reduced risk of orofacial cleft [22] and Down syndrome [23,24]. Interestingly, in Liu et al.’s study [19], the risk of NTDs associated with the variant A allele was only observed in pregnancies of women that did not take folic acid supplements. 

We hypothesised that the BHMT c.716G>A polymorphism impedes the conversion of betaine to dimethylglycine during pregnancy. We tested how plasma folate status affects plasma betaine and dimethylglycine according to BHMT c.716G>A genotype throughout pregnancy. 

## 2. Experimental Section

### 2.1. Participants

A total of 612 women that were recruited before November 2014 from the pregnancy phase of the Reus-Tarragona Birth Cohort (RTBC) were studied. The RTBC is an observational longitudinal study of maternal nutritional status and pregnancy that is being carried out by the Area of Preventive Medicine and Public Health, Universitat Rovira i Virgili and the Areas of Obstetrics and Gynaecology of the University Hospitals: Sant Joan, Reus and Joan XXIII, Tarragona (Spain). The study design and participant recruitment have been previously described [12]. Briefly, women attending their first antenatal visit with a viable singleton pregnancy confirmed by ultrasonography that provided a fasting blood sample at or before the 12th gestational week (GW) were eligible to participate in the study. Exclusion criteria included chronic diseases, surgical interventions affecting nutritional status or medication affecting folate or cobalamin metabolism. The study was carried out with ethical approval from the ethics committees of both participating hospitals and the research has been registered at Clinical Trials. gov: NCT01778205. All participants were informed of the nature and aims of the study and provided signed consent.

Participant age and body mass index (BMI) were recorded at the first antenatal check-up. Data regarding lifestyle, habits and supplement use from periconception throughout pregnancy was collected by the study team using questionnaires at 20 and 32 GW. Socioeconomic status of the participants was defined as low, middle or high according to the family unit income, education level and occupation of both parents [25]. Participants were classified as smokers throughout pregnancy, smokers during the first trimester and non-smokers based on first and second trimester as well as cord plasma cotinine concentrations (see biochemical determinations). Since plasma cotinine is indicative of recent smoking data from questionnaires and prenatal check-ups regarding declared smoking activity by the participants was also considered. 

In line with the Spanish Obstetrics and Gynaecology Society recommendations [26], women were advised to take daily prenatal supplements containing 400 µg folic acid during the first trimester. The specific supplements recommended for this study were composed of 400 µg folic acid and 2 µg cyanocobalamin. 

### 2.2. Blood Sample Collection and Biochemical and Genetic Determinations 

Fasting blood samples were collected at ≤12, 15, 24–27 and 34 GW and nonfasting samples on admission to hospital with confirmed labour and from the umbilical cord. Maternal blood was drawn from the antecubital vein, and cord blood from the vein, into vacutainers containing EDTA-K_2_. Samples were kept at 4 °C for a maximum of 1 h before separating plasma of which aliquots were immediately stored at −80 °C in the Institut d’Investigació Sanitària Pere Virgili biobank (Reus (Tarragona), Spain). Leukocytes were isolated from the blood cells remaining after plasma separation and DNA extracted from these using the Puregene DNA extraction kit (Gentra Systems, Minneapolis, MN, USA). Samples were transported on dry ice to Bevital (www.bevital.no, Bergen, Norway), carrying out biochemical analyses in batches comprising complete pregnancies within 18 months of collection. Plasma folate was determined by microbiological assay with *Lactobacillus casei* [27]. Plasma concentrations of choline, betaine, dimethylglycine, total homocysteine (tHcy) were measured by liquid chromatography–tandem mass spectrometry [28,29]. Plasma cotinine, which is an indicator of recent nicotine exposure, was also determined by liquid chromatography–tandem mass spectrometry [30] and plasma creatinine by modified Jaffé method (Química Clínica Aplicada SA, Amposta, Tarragona, Spain). Classification as current smoker was based on plasma cotinine >10 ng/mL at ≤12 GW (first trimester smoker) and 24–27 GW or in the cord (smoker throughout pregnancy). The maternal BHMT c.716G>A SNP was determined by Matrix-Assisted Laser Desorption/Ionization Time-Of-Flight Mass Spectrometry (MALDI-TOF/MS) as previously described [31].

### 2.3. Statistical Analysis

Previously, we reported a shift in folate status between early and mid-late pregnancy in line with the overall cessation of folic acid supplement use or change in supplement type between the first and second or third trimesters of pregnancy [12]. To account for change in plasma folate status during pregnancy, and to classify plasma folate status according to WHO criteria [32], participants were classified into possibly deficient (plasma folate ≤ 13.4 nmol/L) or normal-high (plasma folate > 13.4 nmol/L) status categories during early pregnancy (based on plasma folate at ≤12 or 15 GW) and again during mid-late pregnancy (based on plasma folate at 24–27 or 34 GW). In the event of falling into different categories at different time points within the corresponding phase of pregnancy, normal-high status was assigned during early pregnancy and the predominant status out of the 3 time points was assigned in mid-late pregnancy. In any analysis using plasma folate status, the category occurring during the corresponding phase of pregnancy was used.

Natural log transformation was applied to normalise the distribution of plasma variables as required for the application of parametric tests. Thus, geometric means (95% CI) are reported for plasma folate, betaine, choline, dimethylglycine and tHcy. In all other cases medians (25th percentile, 75th percentile) are reported and frequencies are reported as % (95% CI). Proportions were compared using the chi-square test and the same test was also used to test the Hardy Weinberg equilibrium of the observed allele frequencies.

Plasma folate, choline, betaine, dimethylglycine and dimethylglycine/betaine ratio and tHcy were compared between the different BHMT c.716G>A genotypes at each gestational period and in the cord by ANCOVA adjusting for gestational age (weeks) at the time of the blood sample, and plasma folate status (in all models except for plasma folate models). The dimethylglycine models were also adjusted for plasma betaine. Interaction between plasma folate status and BHMT c.716G>A genotype in their effects on plasma dimethylglycine, dimethylglycine/betaine ratio and tHcy was tested and if observed, stratified analysis according to folate status was performed. 

Changes in plasma concentrations of folate, choline, betaine, dimethylglycine, the dimethylglycine/betaine ratio and tHcy during pregnancy were assessed using 2-factor repeated measures ANOVA (General linear model; Intrasubject factor time of pregnancy and intersubject factor BHMT c.716G>A genotype) with posthoc Bonferroni correction of *p* values to account for multiple comparisons. The first trimester time point was used as the reference. 

Multiple linear regression analysis was used to investigate the effects of plasma folate status and of maternal BHMT c.716G>A genotype on plasma dimethylglycine at each stage of pregnancy and in the cord. The models were adjusted for plasma betaine and gestational age at corresponding time of blood sample. The usual diagnostic techniques for multiple linear regression analysis were applied (Cook’s distance to identify influential cases and analysis of residuals). Interaction between plasma folate status and BHMT c.716G>A genotype was also tested. All analyses were carried out with SPSS (SPSS Inc., Chicago, IL, USA) for Windows, version 22.0.

## 3. Results

**Figure 1:**
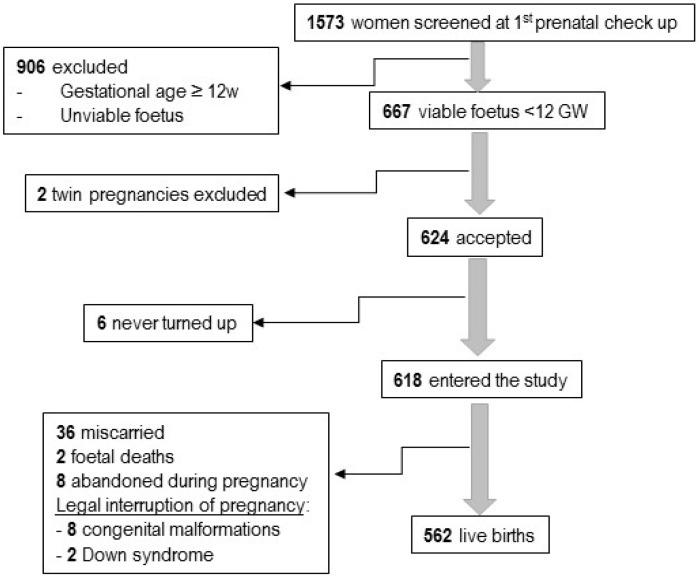
Flow chart of participation in the study.

Participation in the study by eligible candidates and completion (defined as live birth) is illustrated in Figure 1. Of the women screened at their first prenatal check-up, 42.4% were eligible to participate in the study. Of the 624 participants that entered the study (93.8% of those eligible) first trimester data was collected from 612 and 562 went on to have live births. 

Participant lifestyle, plasma folate, cobalamin, choline, tHcy and obstetrical characteristics are summarised in Table 1. This data shows that 80.9% of the women reported planning their pregnancy and 34.1% took folic acid supplements before becoming pregnant. Active smoking during the first trimester and throughout pregnancy was observed in 28.1% and in 17% of the participants respectively. The frequency of the BHMT c.716A allele was 30.8% (95% CI: 28.3, 33.5) and the genotypes were in Hardy Weinberg equilibrium (chi square for observed compared to expected genotype frequencies: 1.64).

Plasma folate, choline, betaine and dimethylglycine concentrations, dimethylglycine/betaine ratios and tHcy at each time point of pregnancy and in the cord, according to maternal BHMT c.716G>A genotype are reported in Table 2. Plasma folate, choline, betaine or tHcy did not differ among genotypes at any time of pregnancy. Lower plasma dimethylglycine was observed in the heterozygote compared to homozygote common genotype at ≤12 GW and at labour. This was also true for the homozygote variant genotype during late pregnancy. A lower plasma dimethylglycine/betaine ratio was observed in the homozygote variant compared to heterozygote genotype at ≤12 GW and compared to the homozygote common genotype at 34 GW. This was also true for the heterozygote compared to the homozygote common genotype at 15 GW. Further adjustment of the ANCOVA models for plasma creatinine and smoking habit did not alter the results.

Plasma betaine gradually decreased in all of the genotypes throughout pregnancy. Plasma dimethylglycine fluctuated in a U shape pattern in the homozygote common and heterozygote genotypes where end of pregnancy concentrations were higher than in early pregnancy. However, in the homozygote variant genotype, plasma dimethylglycine concentrations remained lower than in early pregnancy, throughout pregnancy. The plasma dimethylglycine/betaine ratio gradually increased during pregnancy in all genotypes. 

Interactions between plasma folate category and the effect of BHMT c.716G>A genotype on dimethylglycine were observed at ≤12 and 15 GW and on the dimethylglycine/betaine ratio at ≤12 GW.

**Figure 2:**
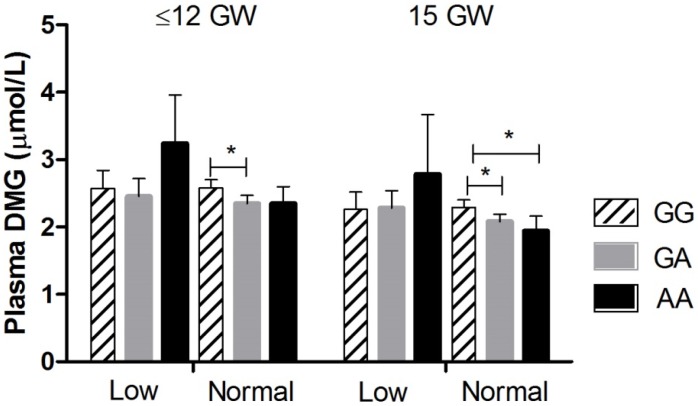
Plasma dimethylglycine according to BHMT c.716G>A genotype and folate status in early pregnancy. G: guanosine; A: adenosine; GW: Gestational weeks. Low: possibly deficient (plasma folate ≤ 13.4 nmol/L). Normal: normal-high (plasma folate > 13.4 nmol/L). At ≤12 GW, Low: GG (*n* = 57), GA (*n* = 48), AA (*n* = 14); Normal: GG (*n* = 231), GA (*n* = 193), AA (*n* = 47). At 15 GW, Low: GG (*n* = 37), GA (*n* = 40), AA (*n* = 6); Normal: GG (*n* = 169), GA (*n* = 141), AA (*n* = 34). The triple screening blood sample at 15 GW is optional and blood samples were available from less participants. Values are least square geometric means. Error bars represent 95% confidence interval. Comparisons between genotypes were made using ANCOVA adjusting for plasma betaine and gestational age at time of blood draw with posthoc Bonferroni correction for multiple comparisons of *p* values: * *p* < 0.05.

A stratified analysis of plasma dimethylglycine for each genotype according to plasma folate status in early pregnancy is illustrated in Figure 2. In the normal-high category of plasma folate status, lower plasma dimethylglycine was observed in the heterozygote compared to the homozygote common genotype at both ≤12 and 15 GW and in the homozygote variant compared to homozygote common genotype at 15 GW.

The associations between plasma folate status and BHMT c.716G>A genotype with plasma dimethylglycine throughout pregnancy and in the cord are reported in Table 3. Low compared to normal-high plasma folate status was positively associated with plasma dimethylglycine throughout pregnancy and in the cord. Compared to the homozygous common BHMT GG genotype, both variant GA and AA genotypes were associated with lower plasma dimethylglycine during early and late pregnancy. The observed associations were stronger in the case of the homozygote AA variant genotype during late pregnancy and in the cord. Further adjustment of the multiple linear regression models for plasma creatinine and smoking habit did not alter the results.

## 4. Discussion

### 4.1. Principal Findings

This study reports for the first time that the A-allele of the BHMT c.716G>A SNP is associated with lower plasma dimethylglycine during pregnancy and in the cord. Low folate status, on the other hand is associated with higher plasma dimethylglycine during mid and late pregnancy and in the cord. Folate status and genotype interacted during early pregnancy and stratification by plasma folate status category showed that the effect of the variant BHMT c.716A allele on dimethylglycine was limited to women with normal-high folate status. By late pregnancy, the variant allele’s effect was independent of folate status. 

### 4.2. Comparison with Previous Studies and Interpretation

We observed similar frequencies of the BHMT c.716G>A genotypes as previously reported in studies from Europe [14,17,33]. 

We interpret the lower plasma dimethylglycine concentrations observed in the presence of the variant BHMT c.716A allele to mean that the conversion of betaine to dimethylglycine is low in pregnant women carrying the A-allele. A similar observation was previously reported in a population study [17]. On the other hand, the higher plasma dimethylglycine concentrations when folate status is low, suggests BHMT upregulation when MTR activity is reduced. In early pregnancy when MTR activity was not reduced by low folate availability, the effects of lower BHMT activity in the presence of the BHMT c.716 A-allele are evident. We hypothesised that this effect would be more pronounced at mid-late pregnancy and with low folate status when the BHMT pathway is more active [12]. Our results showed decreased betaine to dimethylglycine conversion in the variant A-allele carriers, but in early pregnancy this effect was limited to women with normal-high folate status. In vitro experiments have shown that BHMT expression and activity may be modified by the molecular environment. SAM has been shown to inhibit BHMT transcription in human cells [34,35] and SAM [36] and to a lesser extent SAH [36,37] to inhibit BHMT activity in rat liver extracts. Methionine has also been shown to inhibit BHMT [38,39]. A study in MTHFR-deficient mice supplemented with very high doses of folic acid showed that despite greater betaine utilisation, likely for homocysteine remethylation, it was insufficient to maintain SAM concentrations [40]. Despite the differences in molecular environment between *in vivo* and *in vitro* studies, they shed light on potential mechanisms that may lead to our observed effects. A speculative suggestion from our data is that high folate status can lead to BHMT inhibition, with the inhibitory effect being stronger on the variant enzyme. Some studies have investigated the effect of folate on BHMT. Liver *BHMT* expression was reported to be lower in the offspring of rat dams supplemented with folic acid during pregnancy [41] but was unaffected by excessive folic acid intake in mice [40]. It has also been suggested that betaine is spared when folate status is replete [42]. It is possible that the combination of both mechanisms, replete folate status and the variant allele, lead to reduced conversion of betaine to dimethylglycine. Cessation of prenatal folic acid supplementation at the end of the first trimester was associated with a sharp reduction in folate status, and from mid pregnancy the genotype effect on dimethylglycine was independent of folate status. 

No differences in plasma betaine concentrations between the different genotypes were observed. This is not surprising because a stable betaine pool is maintained even when the BHMT pathway is upregulated [9,43], possibly to spare betaine for its principal functions as an osmolyte [44] and in protein stabilisation [45]. 

### 4.3. Implications

BHMT expression and activity in foetal livers has been reported to increase with gestational age in human [5,46] and pig studies [47] which is in line with increased BHMT activity in late pregnancy that we suggest here. Interestingly, foetal liver MTR activity decreases in the third trimester [5]. This supports the idea of complementarity between the BHMT and MTR pathways in late pregnancy and our observations indirectly support this hypothesis. 

Here we show that the BHMT c.716G>A polymorphism, previously associated with foetal developmental defects, affects the BHMT pathway during pregnancy. In situations of upregulation of the MTR and BHMT pathways, such as in pregnancy, the effect of the SNP on BHMT activity may become more evident. Anomalies in homocysteine remethylation affect homocysteine homeostasis as well as methyl group supply to essential epigenetic reactions. Elevated maternal homocysteine has been associated with pregnancy complications affecting both maternal and foetal health as well as foetal development [48] and with lasting developmental effects into childhood [49]. Inhibition of BHMT has been shown to cause hyperhomocysteinaemia in mice [50]. We did not observe an effect of the variant allele on homocysteine. However, the extent of the effect of the SNP on BHMT activity in humans is unknown. Pregnancy itself has a profound effect on homocysteine [51] and may mask any potential effects of the SNP. Comparison of associations between betaine and homocysteine before and after the implementation of mandatory fortification of flour with folic acid in the USA showed that the inverse correlation reported in Framingham study participants with low folate status prior to fortification, was no longer observed post-fortification [52].

Potential modification of effects of folate status by the BHMT c.716G>A SNP should be considered when contrasting/interpreting results from fortified versus non-fortified populations or supplement versus non-supplement users. In addition, the importance of the BHMT pathway in homocysteine homeostasis may be less important in folate-replete populations. 

### 4.4. Strengths and Limitations

A strength of this study is its longitudinal design and follow up from very early pregnancy. It was carried out in the absence of mandatory fortification with folic acid and therefore the effects of prenatal folic acid supplement use were observed. Samples were processed according to specific protocols to prevent artefacts in plasma analyte concentrations caused by temperature and time before separation of blood cells [53].

When folic acid supplementation ceased there was a large drop in plasma folate as we reported previously [12]. Sample power may have been limited in analyses of the effect of the BHMT c.716AA genotype during early pregnancy. However many significant effects of this genotype on dimethylglycine were observed in mid-late pregnancy and in the cord. Another potential limitation to the study was the difference in folic acid supplement use between women. They were recommended to take prenatal supplements containing 400 µg of folic acid during the first trimester. However some took other brands of folic acid containing higher doses or extended their folic acid use beyond the first trimester. We dealt with this in our analysis by classifying the women into plasma folate status categories in early and again in mid-late pregnancy to account for the difference in status that we expected to arise due to differences in patterns of supplement use. 

Although there is increasing evidence that BHMT plays an important role in one carbon metabolism many aspects regarding the mechanisms involved are unclear. Information regarding the effect of the common BHMT c.716G>A polymorphism on BHMT activity and how it is affected by variations in molecular environment is lacking. 

## 5. Conclusions

We conclude that dimethylglycine during pregnancy is affected by both folate status and the BHMT c.716G>A polymorphism. 

## Figures and Tables

**Table 1:** Participant characteristics (*n* = 612).

Age (Year) ^1^		32.0 (29.0, 35.0)
Body mass index (kg/m^2^) ^1^		23.0 (20.9, 25.4)
Planned pregnancy ^2^		80.9 (77.3, 84.0)
Previous pregnancy ^2^		52.6 (48.6, 56.5)
Socioeconomic status ^2,3^	High	43.6 (39.6, 47.6)
	Mid	49.6 (45.5, 53.6)
	Low	6.8 (5.1, 9.1)
Smoking during pregnancy ^2^	First trimester	28.1 (24.7, 31.8)
	Throughout pregnancy	17.0 (14.2, 20.3)
Folic acid supplement use ^2^	Preconception	34.1 (30.2, 38.2)
	First trimester	93.8 (91.5, 95.4)
	Mid-late pregnancy	53.9 (49.6, 58.1)
BHMT c.716G>A ^2^	GG	48.9 (45.0, 52.9)
	GA	40.5 (36.6, 44.4)
	AA	10.6 (8.4, 13.3)

G: guanosine; A: adenosine. Values are ^1^ median (25th percentile, 75th percentile), ^2^ % (95% confidence interval); ^3^ based on total income, occupation and education level of both parents.

**Table 2:** Plasma folate, choline, betaine, dimethylglycine and tHcy during pregnancy and in the cord according to maternal BHMT c.716G>A genotype.

	BHMT c.716G>A Genotype	≤12 GW [546] ^1^	15 GW [440]	24–27 GW [500]	34 GW [485]	Labour [478]	Cord [465]
Folate (nmol/L)	GG	26.8 (24.5, 29.2)	25.6 (23.3, 28.1)	13.3 ^a^ (12.2, 14.6)	10.9 ^a^ (9.9, 12.1)	10.8 ^a^ (9.7, 11.9)	23.9 (22.2, 25.7)
	GA	25.5 (23.2, 28.0)	24.6 (22.3, 27.1)	12.9 ^a^ (11.7, 14.2)	10.7 ^a^ (9.7, 11.9)	10.8 ^a^ (9.7, 12.1)	24.2 (22.4, 26.1)
	AA	24.5 (20.2, 29.6)	25.4 (20.8, 31.1)	12.6 ^a^ (10.3, 15.3)	12.0 ^a^ (9.6, 15.1)	10.2 ^a^ (8.2, 12.9)	22.6 (19.1, 26.6)
	*ANCOVA ^3^ models*	NS	NS	NS	NS	NS	NS
Choline (µmo/L)	GG	7.6 (7.4, 7.9)	7.7 (7.5, 7.9)	9.1 ^a^ (8.9, 9.4)	10.3 ^a^ (10.1, 10.6)	11.7 ^a^ (11.3, 12.1)	28.1 (26.8, 29.5)
	GA	7.7 (7.4, 7.9)	7.7 (7.5, 8.0)	9.2 ^a^ (9.0, 9.5)	10.4 ^a^ (10.1, 10.7)	11.7 ^a^ (11.3, 12.2)	28.9 (27.5, 30.4)
	AA	7.6 (7.2, 8.1)	8.0 (7.5, 8.5)	9.5 ^a^ (9.0, 10.1)	10.5 ^a^ (9.9, 11.2)	11.6 ^a^ (10.7, 12.5)	29.8 (26.8, 33.1)
	*ANCOVA ^3^ models*	NS	NS	NS	NS	NS	NS
Betaine (µmol/L)	GG	20.9 (20.2, 21.6)	14.6 ^a^ (14.2, 15.1)	12.8 ^a^ (12.4, 13.1)	12.8 ^a^ (12.5, 13.1)	13.1 ^a^ (12.7, 13.5)	24.7 (24.0, 25.4)
	GA	21.8 (21.0, 22.6)	15.3 ^a^ (14.8, 15.8)	13.1 ^a^ (12.7, 13.4)	13.3 ^a^ (12.9, 13.7)	13.4 ^a^ (13.0, 13.8)	25.1 (24.3, 25.8)
	AA	20.2 (18.8, 21.8)	14.5 ^a^ (13.6, 15.5)	12.7 ^a^ (11.9, 13.4)	13.0 ^a^ (12.3, 13.8)	12.7 ^a^ (11.9, 13.5)	25.3 (23.8, 27.0)
	*ANCOVA^3^ models*	NS	NS	NS	NS	NS	NS
Dimethylglycine (µmol/L)	GG	2.58 (2.47, 2.69)	2.29 ^a^ (2.19, 2.39)	2.22 ^a^ (2.12, 2.33)	2.51 (2.39, 2.64)	3.00 ^a^ (2.84, 3.18)	3.73 (3.55, 3.92)
	GA	2.37 (2.26, 2.48) *	2.12 ^a^ (2.02, 2.22)	2.11 ^a^ (2.00, 2.22)	2.39 (2.27, 2.52)	2.69 ^a^ (2.53, 2.86) *	3.54 (3.36, 3.73)
	AA	2.50 (2.28, 2.74)	2.08 ^a^ (1.88, 2.30)	2.02 ^a^ (1.82, 2.24)	2.01 ^a^ (1.79, 2.25) **^,†^	2.44 (2.16, 2.76) **	3.29 (2.95, 3.67)
	*ANCOVA ^4^ models*	*p* = 0.026	*p* = 0.040	NS	*p* = 0.002	*p* = 0.002	NS
	Folate genotype interaction	*p* = 0.023	*p* = 0.058	NS	NS	NS	NS
Dimethylglycine/betaine	GG	0.13 (0.12, 0.14)	0.17 ^a^ (0.16, 0.18)	0.19 ^a^ (0.18, 0.21)	0.22 ^a^ (0.20, 0.24)	0.26 ^a^ (0.23, 0.28)	0.17 (0.15, 0.18)
	GA	0.12 (0.11, 0.13)	0.15 ^a^ (0.14, 0.16) *	0.18 ^a^ (0.16, 0.19)	0.20 ^a^ (0.18, 0.22)	0.23 ^a^ (0.21, 0.26)	0.15(0.14, 0.16)
	AA	0.15 (0.13, 0.17) ^†^	0.15 (0.13, 0.17)	0.18 ^a^ (0.15, 0.22)	0.16 (0.12, 0.20) *	0.22 ^a^ (0.17, 0.27)	0.14 (0.11, 0.16)
	*ANCOVA ^5^ models*	*p* = 0.009	*p* = 0.018	NS	*p* = 0.020	NS	*p* = 0.044
	Folate genotype interaction	*p* = 0.038	NS	NS	NS	NS	NS
Total homocysteine (µmol/L)	GG	5.2 (5.1, 5.4)	4.5 ^a^ (4.4, 4.7)	4.6 ^a^ (4.5, 4.8)	5.3 (5.1, 5.4)	6.2 ^a^ (6.0, 6.5)	4.8 (4.7, 5.0)
	GA	5.3 (5.1, 5.4)	4.4 ^a^ (4.3, 4.6)	4.7 ^a^ (4.5, 4.8)	5.4 (5.2, 5.6)	6.2 ^a^ (5.9, 6.4)	4.9 (4.7, 5.2)
	AA	5.3 (5.0, 5.6)	4.5 ^a^ (4.3, 4.8)	4.6 ^a^ (4.3, 4.9)	5.2 (4.9, 5.6)	6.0 ^a^ (5.6, 6.5)	4.9 (4.5, 5.3)
	*ANCOVA ^4^ models*	NS	NS	NS	NS	NS	NS

G: guanosine; A: adenosine; GW: gestational weeks; NS: non-significant. ^1^ *n* varies between time points due to participant loss due to complications, non-attendance of programmed blood draw or delivery elsewhere or failure to collect blood samples in the labour ward; ^2^ values are least square geometric means (95% confidence interval); ^3^ adjusting for plasma folate status and gestational age at time of blood draw; ^4^ adjusting for plasma folate status, plasma betaine and gestational age at time of blood draw. ANCOVA Bonferroni posthoc: * *p* < 0.05, ** *p* < 0.01 versus GG; ^†^ *p* < 0.05, versus GA; Two-factor repeated measures ANOVA versus ≤12 GW (intrasubject factor: gestational age; intersubject factor: BHMT c.716G>A genotype) followed by post hoc Bonferroni correction for multiple comparisons: ^a^ *p* < 0.001.

**Table 3:** Change in plasma dimethylglycine throughout pregnancy according to plasma folate category, and BHMT c.716G>A genotype.

		Model ^1^		Plasma Folate Category ^2^	BHMT c.716G>A Genotype	
		*R*^2^	F (*n*)	Possibly deficient vs. normal-high	GA vs. GG	AA vs. GG
Early	≤12 GW	15.4	22.5 (592) ***	0.07 (0.04) ^3,^*	−0.08 (0.03) **	−0.04 (0.05)
	15 GW	12.0	12.6 (427) ***	0.07 (0.04) ^#^	−0.08 (0.03) *	−0.11 (0.06) ^†^
Mid-late	24–27 GW	11.6	14.3 (507) ***	0.13 (0.03) ***	−0.06 (0.04)	−0.10 (0.06)
	34 GW	18.9	23.8 (492) ***	0.20 (0.04) ***	−0.06 (0.04)	−0.23 (0.06) ***
	Labour	13.2	15.5 (477) ***	0.15 (0.04) ***	−0.11 (0.04) *	−0.21 (0.07) **
	Cord	5.0	5.7 (447) ***	0.08 (0.04) *	−0.05 (0.04) *	−0.13 (0.06) *

F: analysis of variance F-test of overall significance; G: guanosine; A: adenosine; GW: Gestational weeks. ^1^ Multiple linear regression analysis: dependent variable plasma dimethylglycine. Adjusted for plasma betaine and gestational age at time of blood draw; ^2^ Pregnancy (possibly deficient: ≤13.4 nmol/L; normal-high: >13.4 nmol/L); ^3^ β coefficient (standard error of the mean). * *p* < 0.05, ** *p* < 0.01, *** *p* < 0.001, ^†^ *p* = 0.055, ^#^ *p* = 0.087.
